# Stereotyped, automatized and habitual behaviours: are they similar constructs under the control of the same cerebral areas?

**DOI:** 10.3934/Neuroscience.2020010

**Published:** 2020-05-27

**Authors:** Tiziana M. Florio

**Affiliations:** Department of Life, Health and Environmental Sciences, University of L'Aquila, Italy

**Keywords:** basal ganglia, stereotypies, automation, habits, behaviour, intelligence

## Abstract

Comprehensive knowledge about higher executive functions of motor control has been covered in the last decades. Critical goals have been targeted through many different technological approaches. An abundant flow of new results greatly progressed our ability to respond at better-posited answers to look more than ever at the challenging neural system functioning. Behaviour is the observable result of the invisible, as complex cerebral functioning. Many pathological states are approached after symptomatology categorisation of behavioural impairments is achieved. Motor, non-motor and psychiatric signs are greatly shared by many neurological/psychiatric disorders. Together with the cerebral cortex, the basal ganglia contribute to the expression of behaviour promoting the correct action schemas and the selection of appropriate sub-goals based on the evaluation of action outcomes. The present review focus on the basic classification of higher motor control functioning, taking into account the recent advances in basal ganglia structural knowledge and the computational model of basal ganglia functioning. We discuss about the basal ganglia capability in executing ordered motor patterns in which any single movement is linked to each other into an action, and many actions are ordered into each other, giving them a syntactic value to the final behaviour. The stereotypic, automatized and habitual behaviour's constructs and controls are the expression of successive stages of rule internalization and categorisation aimed in producing the perfect spatial-temporal control of motor command.

## 1. Introduction

Human behaviour expresses through a large range of movements from simpler reflex to complex and purposeful motor acts. Automatic, innate behaviours are structurally organised and functionally available as complex reflex in the newborn [1]. Spinal and central patterns generators are arranged as innate circuits and, once functionally activated, they give raise to stereotyped and automatic motor behaviours strictly dependent from stimuli [2]. Moreover, the ability to recognize the orthostatic locomotor progression [3],[4] and the face gestures [5] are greatly innate and shared by all ethnic groups and human cultures [6],[7]. Specific innate circuits appear to be evolved, although they have been conserved and replicated in basic physiological mechanisms through different phyla from lampreys to vertebrates and mammals [8]. If some functional features of motor control can be found in a variety of vertebrate model systems, it means that genetic mechanisms had repeatedly fixed the results of newly emerging behaviours resulting from the reorganisation of neural networks, thus confirming the usefulness of such strategy through different organisms [1]. This is the case of Basal Ganglia (BG), where basic modules and functional design are thought to be at the basis of exaptation, that means the mechanism acting on control and expression of newly emerging patterns of behaviours [9]. On the other hand, the successful emergent functions are stored in the cerebral cortex and cross-distributed in different areas to be simultaneously elicited during decision-making activity [10].

Therefore, the structural organisation of the spinal reflex chain and central patterns generators systems can be thought as firstly conveying input and output signals and then organising the primarily instinctive behaviours in greater complexity [2]. The first step in the categorisation of such a complexity can be found in the stimulus-response association enabling the learning of information between the specific motor acts and the related stimuli [11],[12]. Impairments of such automatic motor behaviours, on the other hands, are lost when specific midbrain neural centres are strongly compromised as in neurodegenerative pathologies, like Parkinson's disease (PD) [13],[14]. Through the action-outcome association [15], reciprocal top-down and bottom-up synergies act in upgrading behavioural motor acts [1],[16] with goal-directed movements that are sensitive to both the value and contingency of external or internal stimuli. In other words, from newborn to adulthood development the environmental, motivational and memory derived information processing synergistically contribute to select the best motor acts whenever significant inner/outer cues reach the threshold to move, and it goes on during the whole life.

The overall recognised model of BG function is based on the parallel pathway processing through which cortical-basal ganglia-thalamic-cortical information are subjected to opposite effects, providing the neural mechanism to rapidly switch from planned to alternative movements [17]. Habits, stimulus-response associations, instrumental behaviour, procedural learning, goal-directed actions, reinforcement learning, are terms identifying neural process, all implicating BG mediation, that involve a contingency on stimuli [18],[19]. On the contrary, the movements elicited by fixed action patterns, the reflexes, are independent of behavioural consequences [16]. In between, the automation of learned motor acts is based on the acquisition and internalization of rules linking events, so that motor and cognitive skills can be expressed in a similar way to the stereotypic execution of a reflex requiring no attentive cost [20].

Here, neglecting the positive or negative motivational drive of stimuli, we limit our analysis to the rule function in classifying the interaction between the subject, emitting actions, and the environment, providing stimuli; of utmost interest being the mechanistic process whereby the external stimuli become internalised. Against such a backdrop, the Ventral Tegmental Area-Hippocampus-ventral striatum pathway [21],[22] and could be seen as part of a dynamical loop where the dopaminergic and cholinergic modulation plays a crucial role, both during the recognition of the stimulus (becoming significant) and the early memory processing [23]. Acting as a gateway system, BG dopaminergic mediated pathway could convey the time-scaled organisation of motor control fixing rules as spatial-related or habit-referred [24],[25]. The reciprocal cholinergic innervation connecting the Nucleus Basalis of Meynert with the marginal division of the ventral striatum [26], on the other way, may represents the anatomical connection between the explicit and implicit memory systems, as already been hypothesised [27] and furnish the circuitry relating the repetitive patterns of behaviour observed in various psychiatric disorders [28], and after psychomotor stimulant administration [29].

Then, the present work, far from being a comprehensive review of human behaviour, is focused in evaluating BG functionality in the rule-based generation and control of the behavioural categories. To this aim, only selected recent advances are proposed as start point to analyse motor categories useful for modelling and theoretical construct of motor acts when depicting intelligent behaviour and consciousness. The proposed executive BG motor control [30],[31], consisting in the ability to detect rules between events, can provide a conceivable framework between motor impairments and psychiatric disorders [32]. This being a useful tool in selecting effective pharmacological approaches and physical activities that could significantly improve brain plasticity and cognitive functions [33]–[35].

## 2. Defining rules

Rules shape simple and complex behaviours. The execution of a complex motor sequence requires the control of two main aspects concerning movement management: the spatial pattern of muscular activation and the exact timing of each sub movement. Here the rules are represented by the target representation, the target reaching strategy, and the final control of the reached target [25],[36]–[38]. Habits, on the contrary, are represented by well-learned motor scheme no more depending from external signals to be operated [39]. Spatial and temporal variables have already been selected and acted to obtain the best performance. The best accomplishment is the only rule. This kind of motor performance is so highly automatized to be stimulus-response dependent similarly to a motor reflex in which the stimuli had been internalised.

The ability to shift between internally and externally cued movements consists in picking up an existing motor scheme and realising a new pattern of motor acts to satisfy the new rules demands [40]. The rules, here, are represented by the spatial-temporal connection existing through the motor acts and the possibility to rearrange after chunking them [41]–[43].

Through learning and memory storage of rules it is possible to build an imagery representation of movements in which a preview of the entire motor scene, comprehensive of self [44] and outer units of the motor acting and the environment [45] is virtually executed to test the expected outcomes [46],[47].

Depending on the complexity of the motor response, motor processing can be chained into sequences of almost automatically performed single motor acts, anatomically controlled by specific brain areas extending from cortical to spinal neural centres. Each cerebral region summarises its own contribution realising the integrated version of the final motor response. Spinal neurons are organised to rapidly respond to stimuli producing an immediate stereotyped behaviour. Supplementary Motor Area is especially involved in self-initiated, well-learned, complex, and sequential movements, and its functions are more closely related to the timing of movements than spatial programming [20]. The Lateral Premotor cortex is able to automatically recognize the rules underlying each specific motor act [20]. Cues deriving from sighted motor acts are able to evoke specific electrophysiological patterns in premotor neurons, as well as the one expressed when the movement is required to be acted. This neural system makes possible discerning the underlying intention when someone else performs movements [47],[48].

## 3. Defining stereotypic, habitual and automatic behaviours

Exact definitions and boundaries identifying stereotyped, automatized and habitual behaviours are unmet or even opposing each other. Stereotypic behaviours are referred to consist in repetitive motor response sequences lacking any apparent function. They are generally referred as abnormal repetitive movements, characterised by higher frequency and extended duration, able to impair daily life and socialization [38]. Stereotypic behaviours can also be thought as actions lacking a termination phase in which the repetition of the same behaviour sequence anchors the locomotor control within a recurrent (positive) feedback loop. Hypothesising that goal-directed behaviours are composed of both anticipatory/appetitive and terminal/consummatory components, stereotypies can be seen as the re-proposal of the appetitive stage not followed by consummatory phase, lacking the negative feedback and repetition of the truncated sequence [49]. On the other hand, between stereotypic behaviours there are highly organised patterns of sequential movements such as sexual, aggressive and grooming behaviours [50]–[53]. All of them are characterised by syntactic chains of fixed and repetitive action patterns so that once begun they do not require sensory feedback to be completed. The unbalancing of self-grooming behaviour is a validated model of human pathological motor or thoughts repetitive behaviour [53]. The dorsolateral striatal region is essential for the control and coordination of the complex, repetitive and sequential pattern of actions required for the rodent self-grooming execution. The striatal patterning and sequencing capability results in an ordered execution of the motor pattern chain giving them their syntactic value [53]. Moreover, the stimulation of the frontal ventral medial striatum has been found to enhance grooming behaviour, but not repetitive behaviour elicited by type 1 dopaminergic receptor (D1) direct striatal-nigral pathway activation [38].

Automation is the way through which cognitive and motor skills become faster and accurately executed when they are practiced. A compelling study of the underlying mechanisms is reported by Ashby et al. [54]. After the early stage of learning, characterised by a goal-directed behaviour in which the action-outcome relation is acquired, the repetition of such (selected) behaviour could be trained until habits or automatized behaviour is accomplished. Interestingly, novel behaviours processing, which was traditionally thought to be initiated in the cerebral cortex and automatized by transferring control to subcortical structures, has been challenged by evidence that initial learning is accomplished in subcortical structures as well. Specifically, the associative region of the striatum is selectively activated during early learning, whereas the sensorimotor striatal area is active after automaticity has developed [15],[55]–[57]. The activation of the dorsomedial associative area of the striatum is required during initial skills acquisition. On the contrary, the dorsolateral sensorimotor area of the striatum responds much more strongly with extended training, when, at the same time, the associative striatal area decreases its activation [54]. Automaticity has also been defined as a rigid behaviour in which performance requires limited attentional resources involving changes in widely distributed neural networks [58]. Notably, the authors introduce an “intermediate” passage concerning the habit formation. Their proposal is that BG operate in both situation of simple and complex motor responses, inducing the Stimulus–Response dependent habit formation in the first case, and routing toward the cortico-cortical performance the basic relations that permits the representations of rules connecting different events in case of complex motor sequences [58].

Summarising, from a neurophysiological point of view, habit formation occurs as a result of an overtraining process and is characterised by a shift from Response–Outcome to Stimulus–Response learning, corresponding to a dorsolateral shift in the cortico-striatal activity loop [42],[49],[59]. When behaviour becomes automatically elicited by external cues, shifting from Goal-Directed to Stimulus–Response habit performance, striatal activity is correspondingly lifted from dorsomedial to the dorsolateral area [60]. Interestingly, sensorimotor dorsolateral area of the striatum is much more activated when motor performance requires sequences concerning single motor responses [54]. In other words, the Response–Outcome learning to Stimulus–Response shifting corresponds to the internalisation of outcome initially linked to a specific response, so that such a role can be the “new” internalised signal (cue) able to drive the selected response automatically. The processes happening in the striatum during the transition from the initial learning to the automatization of the motor acts, enable the cortico-cortical representations mediating automaticity. During the habit formation, the training elicits the (sequential) ruled execution of the behaviour until it becomes automatic. This transition is characterised by the shift from early learning to intermediate Stimulus–Response performance and then to the correct representation of the motor plan. The representation of the motor plan results from the correct categorization of events relationship and it is arranged into a motor scheme, that can be automatically executed by cortical areas intervention. At that point skills became automatic.

As a first conclusion, learning of motor and cognitive skills consists in the first acquisition and memorization of rules linking different events (objects, movements, stimuli...). Successively to this process, the neural control disentangles from external inputs (cues are internalised) selecting the best motor response that match the evoked target through automation. Response automation is newly entangled to the internalised cue that elicits motor response without any attentive cost, in a similar way to the stereotypic execution of a reflex.

We can extend these considerations by looking at the environmental and motor neural representations. Very recently, it has been demonstrated that dorsomedial striatal cells are able to code for the egocentric coordinates, managing their firing rate in relation to direction and distance from the Open Field-testing walls [61]. Such a neural representation of the environmental boundaries is hypothesised to interact with grid hippocampal cells that are able, in turn, to process the allocentric representation of spatial coordinates. Both grid, border and head direction hippocampal cells and egocentric cells in striatum showed a constant firing rate across different environments [45],[61]. A shifting from hippocampal allocentric representation of the environmental cues into the striatal egocentric, and viceversa, is also supposed [61].

We have very recently investigated body centred and peripheral turning patterns evocative of egocentric (self-centred) and allocentric (external world-centred) spatial signals processing. Interestingly, the progressive evolution of turning behavioural pattern was characterised by a peripheral- to a self-centred transition strongly influenced by dopamine (DA) modulation [62].

Furthermore, it looks useful to refer here that, in memory and learning studies, the dorsolateral striatum exhibited a first appearing cognitive place preference, that was replaced by a habit response preference when a behavioural overtraining was imposed. Interestingly, such a switch was enhanced by post-training glutamate managing of dorsolateral striatum [63]. Mammalian memory is proposed to be organized into multiple systems dissociable by their distinct operating principles and brain locations [27]. Hippocampus-dependent spatial/cognitive memory is dissociable from learning and memory functions sub served by the BG. Evidence suggests that BG may be functionally heterogeneous, with different subregions mediating distinct types of learning and memory [64]–[69]. Lesion and behavioural pharmacology studies in rodents have delineated distinct mnemonic functions for the dorsolateral striatum, dorsomedial striatum, and nucleus Accumbens [60]. The dorsolateral striatum mediates stimulus-response/habit memory. The dorsomedial striatum mediates complex maze learning, cognitive flexibility, and action-outcome learning, and the nucleus Accumbens mediates stimulus-outcome learning and the motivational control of learning and behaviour. Therefore, the BG may incorporate limbic, sensory, and executive information from higher-order brain nuclei to influence motor behaviour and, thus, generate behavioural strategies for gaining favourable outcomes [27]. The coordinated contributions from Hippocampal and the BG-mediated system have been both recognised in spatial processing. The Hippocampus-mediated place preference response acts earlier with respect to the BG response preference strategy, taking place when habit has been structured [70].

At this point, a brief account for BG functional understanding is appropriate.

## 4. BG features

The BG are topographically organized in extensive cortico-striatal loops that can facilitate or suppress action representations in the frontal cortex [71],[72]. Different striatal regions are specifically involved in acquiring and/or expressing different habits, depending on cortical and mesencephalic inputs, as well as on their intrinsic circuitry [73],[74]. In agreement with the participation of the caudate nucleus in associative learning as part of an executive loop [23],[75],[76], neuroimaging studies have reported brain activation in the head of the caudate nucleus during the initial formation of rule-based behaviour [77]. It is known that during the early stages of learning the fronto-striatal circuits are strongly activated, but this activation decreases as stimulus-response association consolidates [78]–[80]. Neuronal activity in the putamen, on the contrary, is thought to be more involved in the execution of well-learned rules [23],[81],[82].

Cortical inputs directly excite striatal output neurons, inducing different forms of long-term plasticity at glutamatergic cortico-striatal synapses [83]–[85]. Notably, the temporal relationship, between the striatal interneurons and cortical activities, is critical for the induction of activity-dependent long-term plasticity [86]–[88] and may efficaciously modulate the basic mechanisms allowing BG to operate in procedural learning and memory [65],[89]–[93]. Via the Supplementary Motor Area, BG provide the mean to switch between new and learned movements, and the internal timing cues facilitating the sub-movements initiation in a well-learned motor sequence [36],[94]. Moreover, BG convey the proactive mechanism to switch from an ongoing movement to another when significant environmental cues are detected [95]. The modular organization of the striatum is coherent with a hierarchical model of cortico-striatal functioning, thus adaptive behaviour is connected to identification (motivation; ventral striatum), planning (cognition; caudate) and implementation (sensorimotor coordination; putamen) of significant goals [96]. Therefore, the switching mechanism through which new, contextually successful, motor sequences are selected and internalised to be executable without external cues (self-driven), represents the way BG first recognise the difference between externally and internally cued movements and then operate their motor control [20].

A fine regulation level of DA is essential for the proper functioning of the BG circuits. DA is involved in BG learning processes, by strengthening or weakening the efficacy of cortico-striatal synapses [97],[98]. An excessive release of DA leads to general disinhibition of motor and other behavioural impulses that facilitate behavioural activation, on the contrary insufficient DA release leads to a general inhibition of movements and impulses. In this way, striatum learns how to react at different patterns of cortical activation. The striatum behavioural-dependent plasticity can operate through the time scheduling differentiation of neuronal firing rate in the specific striatal area. The electrophysiological investigation of the neural activity patterns from the dorsolateral and dorsomedial striatum has been correlated with habitual and automatized performances. In fact, it has been recently described an interestingly dissociation of the sequence-related processing between the early vs the extended training of both striatal areas related to the repetitive execution of an array of movements [99].

Thus, BG machinery provides the cognitive control of behaviours within a continuously changing environment. This strategic task is operated through the acquisition of rules connecting different circumstances, objects and specific events and the selection of the most appropriate motor response. Such a pivotal aptitude in grasping the outcomes linking different events is exploited into habit formation, automatization of complex motor sequences and behavioural switching ability. Besides to avoid computational overload, such important mechanisms are at the basis of the cognitive and motor skills and overall expertise. The associative learning of object, environment and event connection become predictive of other relevant conditions, that can be experienced through the imagery, thus enabling to find strategies and solutions before the factual execution of the movement [49].

Because the overactivity or underactivity of BG-Thalamic connectome results in hypokinetic or hyperkinetic disorders, it is generally accepted that the balanced activation of both direct and indirect pathways is responsible for the production of coordinated movements. Accordingly, through BG and cortico-striatal circuits, motor performances and cognitive results are strictly linked [100]. In fact, the working memory makes available both the motor plans and the goals necessary to perform a sequence of movements [58]. These relationships reveal the importance of cognitive training in PD treatment [33],[34].

Switching between automatic and voluntary controlled movements appears to be the main function of BG [101]. The BG-cortical system can contribute to behavioural flexibility supporting the selection of adaptive responses and to help behaviour modulation in response to task demands [70]. BG dysfunctions can cause stereotypies in which voluntary guided movements are replaced by automatized action syntax triggered by external cues [49],[102]. In particular, D1 mediated direct pathway seems to be primarily involved in repetitive behaviours induction (obsessive compulsive disorders correlated), contrarily to the ventro-medial part of the striatum primarily involved in grooming release [38]. Switching movements throughout the scales of time is the prerequisite to organize motor behaviour in response to significant environmental stimuli.

## 5. Motor, non-motor and psychiatric disorders indications

Looking at different motor impairments deriving from distinct neurological and psychiatric pathologies it is possible to argue that stereotypies, habits and automatic motor performances would be managed by different functional loops. PD and related motor disorders, Tourette syndrome, and psychiatric disorders (autism, obsessive compulsive disorders) are only few examples characterised by the development of compulsively repetitive behaviours, stereotypies and cognitive decay [28],[103]–[106].

PD is an extremely heterogeneous disorder and may be seen as the expression of motor categories regression. Postural reflex disturbances and postural instability, generally occurring later in the clinical evolution of PD, are no more considered as an essential diagnostic feature [106]. However, the probability to develop cognitive disorders is strongly related to the severity of basic (axial) motor control impairments [107]. PD patients are impaired in shifting between different motor paradigms in the early phase and suffer for dysfunctional managing of automatic motor skills in the late stage of disease progression. When required to shift from a highly automatized to a novel motor act, PD patients show increased impairments, probably due to the inaccuracy in substituting the new rules necessary to control the new motor act [108]. In late PD, basic activities, such as the postural control of gait and balance, are compromised with a substantial effect on their QoL. Notably, PD rehabilitation is aimed at relearning motor performances through intensity, repetition, specificity and complexity of motor modules. Practice and training of such modules are completed by focusing on external cues, bypassing BG dysfunctional activity and accessing to the cerebellar ancillary pathway [109]. Postural sway has been showed to be a sensitive measure in detecting early PD progression, thus revealing the BG role in maintaining axial tone and postural control [110]. A recent study of a classical PD rat model confirmed the trend to spontaneously swing toward the lesioned side soon after toxic lesioning [111]. Since axial tone is directly connected to trunk rotation ability, providing a measure for passive resistance to external movements and risk of falling [112], the study of medio-lateral swing is of particular interest being related to the BG shifting from automatic to voluntary locomotor control in PD patients.

## 6. Recent BG investigations

Recently, besides the spatial egocentric representation in the dorsomedial striatum [61], new advances have been done concerning the a cross-hemispheric nigrostriatal DA projection [113]. The projection deriving from the contralateral hemisphere enable the differential DA regulation of striatal functional areas determined by the different distribution of the type 2 dopaminergic receptors (D2). The functional effect of such an inter-hemispheric arrangement enables the DA signalling synchronicity between the two hemispheres in healthy condition. Besides the compensatory effects, a pharmacologically induced over stimulation of the ventral striatum can be hypothesised with rather large effects on striatal plasticity, implications for PD, and pharmacological addition [113].

Coming from Ventral Tegmental Area, the DA innervation in the Hippocampus [21] has been linked with behavioural plasticity. Notably, a decreased efficacy of learning and memory behaviours when the Hippocampus is subjected to DA agonist apomorphine has been recently reported [24]. The ventral striatum collects inputs from the Hippocampus, whereas the dorsal striatum receives inputs from the frontal cortex. The Hippocampal-Ventral Tegmental Area DA loop has been described to mark novel info, differentiating it from the already stored ones. Based on these connections, BG can operate on behavioural flexibility, learning how to change the reinforcement value of behaviours, thus preventing the no longer appropriate actions and endorsing the newly appropriate one [27],[32],[114],[115]. Drug addiction, obsessive compulsive disorders, pharmacologically induced motor impairments can be reviewed as a consequence of altered functionality of the Ventral Tegmental Area-Hippocampus-ventral striatum dynamical loop. In the hypothesis of hyper-DA state [70],[113], resulting from Apomorphine activation of both D1 and D2 receptors, the stimuli (events) novelty would be lost, the comparative function with cortical-driven signal would be impaired, and as a consequence habits and repetitive behaviours would be acted.

The newly described ACh connection existing between the Nucleus Basalis of Meynert and the Marginal division of the ventro-medial portion of the striatum [26] could be indicative of critical cognitive defiance developing when striatal dysfunction involves almost entirely the BG machinery.

Altogether, these data indicate a time-scaled organisation of motor control. Earlier in the behavioural organisation, external cue(s) suddenly elicits simultaneous activity in different cerebral areas, to inform the motor system of the significative content of events. DA mediated loop connecting ventral striatum, Hippocampus and Ventral Tegmental Area is mainly involved during this initial processing. A cross-modal and inter-hemispheric association is then triggered to catch the rules contained in the event signalled by DA as significative. BG are in the perfect location to receive all sensory information and associate them with “marked” motor events, that are enabled to be referred in the egocentric space. Depending on the complexity of the motor-cognitive event, the promoted behaviour can be stored just as a Stimulus-Response habit, or further processed, through the cortical-BG circuitry, to be completely stored as a collection of actions, after the automatization as Outcome-Response Skill. Any impairments affecting the space re-location or timing processing of the external-internal signalling reorganisation can impact the behavioural expression resulting in motor and/or cognitive defiance. Both DA- and ACh-mediated pathways are involved in learning and memory storage of the implicit and explicit contents.

Therefore, when BG input-output are normally managed, stereotypies, habits and automatized movements can be finely controlled, allowing the sequential organization of each behavioural event. Just recently, issues have been pointed on the detection of statistically significant relationships between behavioural ethograms over time in a PD rat model. When analysed at late post-lesion phase, the t-pattern analysis revealed a reduced number of complex ethograms and the altered sequencing of the remaining simpler ones. These data suggest that BG dysfunction manifested on behavioural complexity and variability operating a simplification of behavioural strategies [116].

Motor automaticity can be viewed as the main system enabling cortical areas to focus attention on other procedures [117]. Impairments in motor automaticity require increasingly cortical intervention acting via attentional processes and high computational cost, thus interfering with the motor performance itself (see PD patients). An interestingly study [108] has shown that PD patients are impaired or improved in saccade switching when they were required to overwhelm an automatized movement with a voluntarily triggered one, or vice versa, respectively. Authors posited an executive function for BG mediated task set control, based on rule-representation of the fronto-striatal pathway, resulting in a delayed ability to select an alternative response. Unfortunately, the possible influence of task direction in relation to the clinical symptom's lateralisation was neglected.

## 7. Outcomes

Everyone knows what intelligence is, but it is difficult to exactly define it. An equation [F=T∇Sτ] states that intelligence is a force acting to maximise future freedom of action, as a consequence intelligence correlates with the ability to manage the entropic factor [118]. Then, a starting point to describe intelligence's attributes can be done looking at intelligence as a flexible functional expertise giving (us) the ability to grasp external facts (the events) and re-externalise (through movements) the rules between events that we were able to catch. Concerning the motor intelligence description, in this brief review the discussion has been targeted to such demonstration.

Motor intelligence expresses through (i) motor complexity, (ii) ability to shift, and (iii) habits building. Motor complexity depends on spatial coordination and movement timing [119]. The ability to shift concerns the possibility to change between internally cued movements depending on self-initiated, goal-directed movements vs externally or cue driven movements. Habits building is proposed as the ability to realize the motor control based on the outcome of stimulus-response learning. All of the above approaches expressing motor intelligence are interconnected by the possibility to automatize the complexity and, conversely, by the ability to rationalize habits. In the first case cognitive resources can be released, in the second new mechanisms can be introduced also on a well-established motor control.

The BG function in patterning actions produces an ordered execution of the motor sequences, in which any single movement is linked to each other into an action, and many actions can be ordered to each other, such as to give a syntactic value to the final behaviour [120]. This is the proposed answer, raised in the title of this paper, concerning stereotypic, automatized and habitual behaviour's constructs and controls.

BG functioning is quite inspiring and suggestive for pervasive AI development and applications. The BG are characterized by a modular and gating functioning and exert the best spatial-temporal control of motor command. The way the BG receive and transform motor commands in flexible operative output, appears the best target for AI developments. Many wearable devices have been released, and a flow of new data will be available to the high categorisation of movements ever done before. As this goal will be accomplished, the amazing cerebral ability to produce skills in an emergent manner would be approachable.
